# Physical Assessment of CAD/CAM and 3D-Printed Resin-Based Ceramics Integrating Additive and Subtractive Methods

**DOI:** 10.3390/polym17162168

**Published:** 2025-08-08

**Authors:** Khalid K. Alanazi, Ali A. Elkaffas

**Affiliations:** 1 Conservative Dental Science Department, College of Dentistry, Prince Sattam Bin Abdulaziz University, Alkharj 11942, Saudi Arabia; a.elkaffas@psau.edu.sa; 2 Department of Operative Dentistry, Faculty of Dentistry, Mansoura University, Mansoura 35516, Egypt

**Keywords:** 3D-printed, CAD/CAM, hybrid resin composites, surface roughness, water solubility, water sorption

## Abstract

Additive manufacturing (3D printing) using Computer-Aided Design (CAD) has emerged as a cost-effective alternative to subtractive milling in restorative dentistry, offering reduced material waste and lower production costs. This study aimed to compare the physical properties, specifically water sorption, water solubility, and surface roughness, of milled and 3D-printed hybrid resin composite materials. Standardized disk-shaped samples were fabricated using a digital workflow. The additive group included 15 samples printed with a DLP printer using CROWNTEC resin at three different orientations (0°, 45°, and 90°), with five samples prepared at each printing orientation. The subtractive group consisted of specimens milled from the SHOFU DISK hybrid resin composite. Surface roughness samples were also prepared for both methods. Statistical analysis using one-way ANOVA, post hoc tests, and paired t-tests revealed significant differences among groups in all tested properties (*p* < 0.001). Subtractive manufacturing consistently outperformed additive techniques. Among the printed groups, orientation at 0° showed the most favorable outcomes. Moreover, polishing significantly improved surface roughness in both manufacturing methods (*p* < 0.001). These findings emphasize the influence of the fabrication method and printing orientation on the clinical performance of hybrid resin composites, highlighting the importance of polishing in optimizing the surface quality for 3D-printed restorations.

## 1. Introduction

The ability of CAD/CAM systems to decrease or eliminate the need for human labor and processing time is a major reason for their recent rapid rise in popularity [1]. Casting, modeling, and impression-taking errors could be reduced by comparing CAD/CAM with traditional methods [2]. The two main approaches used in computer-aided design and manufacturing systems are additive and subtractive. The additive method, also known as three-dimensional (3D) printing, is used to create restorations by layering successive layers of porcelain or resin [3]. As a result, this process lowers costs while reducing waste [3]. Milling, or subtractive manufacturing, involves cutting from preformed blocks into the desired shape, which leads to more waste and higher costs for milling tools [3,4].

Using hybrid resin composites with less inorganic filler is necessary for achieving beneficial 3D printing results [5]. Since both 3D-printed resin and hybrid resin composites have lower physical qualities, they are primarily used for intermediate restorations that last longer [5]. New 3D-printed hybrid resin composites containing ceramic fillers offer an option for the long-term replacement of certain teeth [6]. However, before these materials can be recommended for clinical use, more in vitro and in vivo studies are needed to evaluate their long-term physical and mechanical performance [7].

Many different hybrid resin composites have been developed and studied, each with its unique microstructure that influences its clinical use in dental restorations [7,8]. Some research suggests that hybrid resin composites could mimic dentin’s modulus of elasticity. Furthermore, it is possible to expect a more consistent distribution of stress within the system when loading hybrid material restorations [9]. Resin composites incorporated into a polymer matrix can be broadly classified into two types: polymer-infiltrated ceramics and resin nano-ceramics [10]. A high filler particle content in resin nano-ceramics has been studied extensively and shows promising physical properties, especially reduced surface roughness [11,12,13]. Additionally, the distinctive microstructure of polymer-infiltrated ceramic network materials provides the benefit of reduced water sorption and solubility, which is attracting the attention of oral health specialists [14].

This study addresses a critical research gap concerning the lack of comparative data on the physical properties of hybrid resin composite materials fabricated using both additive (3D printing) and subtractive methods. While numerous studies have evaluated either 3D-printed or milled materials individually, limited research has systematically compared these two fabrication techniques using the same material class (hybrid resin composites), particularly focusing on water sorption, water solubility, and surface roughness under standardized conditions and across different 3D printing orientations. Moreover, the effect of printing orientation (0°, 45°, and 90°) on these physical properties remains underexplored, despite its known influence on mechanical performance. By incorporating orientation-specific comparisons and using standardized ISO testing protocols, this study fills a knowledge gap that is essential for guiding material selection and optimizing clinical outcomes in restorative dentistry.

Most research shows that 3D-printed temporary dental crowns and fixed prosthetics have inferior physical qualities compared to their milled CAD/CAM and traditionally processed counterparts, regardless of composition. When compared to polymethylmethacrylate (PMMA) resins milled with CAD/CAM software version 2.0.16003, three studies found that 3D-printed PMMA resins had poor color stability [15,16]. Research by Atria et al. [17] and Tasin et al. [18] indicated that, compared to CAD/CAM-milled PMMA, conventional bis-acrylic provisional resins, traditional PMMA, and 3D-printed hybrid resin composites had poor color stability. Conversely, in two separate studies, 3D-printed hybrid resin composites showed better color stability than bis-acrylic resins and more traditional PMMA [19,20].

Nevertheless, the literature on the long-term durability of milled and 3D-printed hybrid resin composites is still scarce. Therefore, this study aims to evaluate the physical properties of milled and 3D-printed hybrid resin composites that integrate additive and subtractive methods. The null hypothesis posited that there would be negligible variations in surface roughness, solubility, and water sorption of milled and 3D-printed hybrid resin composites integrating additive and subtractive methods.

## 2. Materials and Methods

### 2.1. Study Design

**Figure 1:**
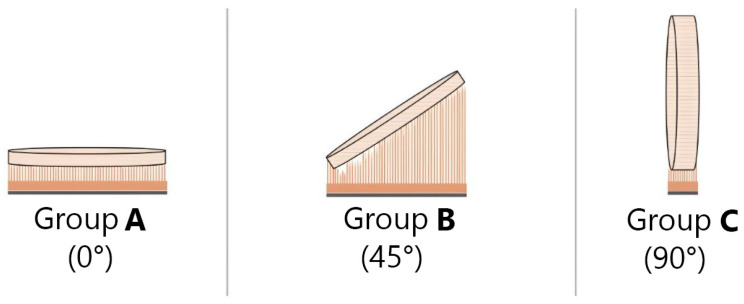
Diagrams showing the direction of load on printed layers and the deposition of 3D-printed groups’ layers are provided by schematic charts: categories A, B, and C, having printing orientations of 0, 45, and 90 degrees, respectively.

An assessment was conducted on two composite resin materials fabricated by distinct methodologies: additive and subtractive. The additive samples were categorized into three categories based on layering direction: 0°, 45°, and 90°, as illustrated in Figure 1. Sample preparation, water sorption, and solubility assessments were conducted in accordance with the ISO 4049 [21] standard, whereas the surface roughness evaluation conformed to established methodologies in the literature.

### 2.2. Sample Preparation

**Figure 2:**
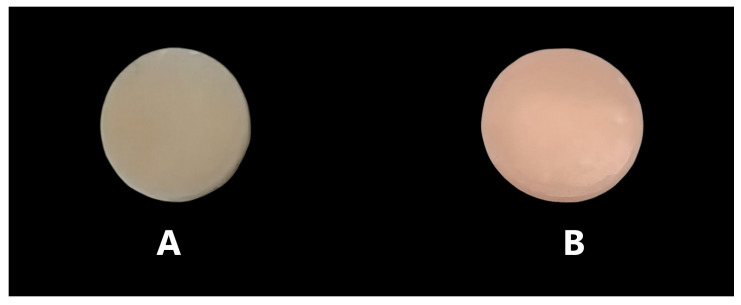
(**A**) 10 mm diameter × 2 mm thick sample for surface roughness test; (**B**) 15 mm diameter by 1 mm thick sample for water sorption and solubility tests.

Using 360 Fusion software version 2.0.16003, a 15 mm diameter by 1 mm thick sample was designed. The design was imported into the 3D printing software (RayWare 2.9.2, SprintRay Inc., Los Angeles, CA, USA) for additive sample preparation. Then, 15 samples were printed with a DLP 3D printer (SprintRay Pro55, Los Angeles, CA, USA) using CROWNTEC resin (Saremco Dental AG, Rebstein, Switzerland), with five samples printed for each orientation (as shown in Figure 1). The design was imported into the Ceramill Mind CAD program (V2.4-7437, Amann Girrbach AG, Mäder, Austria) for subtractive fabrication. A five-axis milling machine (Ceramill Motion 2, Amann Girrbach AG, Mäder, Austria) was used to mill a cylindrical sample (15 mm diameter by 12 mm thick) from a disk of hybrid resin composite (SHOFU DISK HC, SHOFO Inc., Kyoto, Japan). Subsequently, five samples (15 mm diameter by 1 mm thick) were cut from this cylinder (Figure 2). 

The same process was used to create 10 mm diameter × 2 mm thick surface roughness samples (n = 18) for each group (Figure 2). In the next stage, 9 samples from each group were polished using an automated wet polisher (Automata, Jeanwirtz, Dusseldorf, Germany) and silicon carbide grinding paper (600 and 1200 grit, Buehler, Germany) at a wheel speed of 60,000 RPM for 40 s at each step.

### 2.3. Sample Testing

#### 2.3.1. Water Sorption and Solubility

A. Conditioned samples

The dried silica gel was placed in a desiccator to hold the samples in a rack. We transferred the samples to a second desiccator containing recently dried silica gel after placing the first one in an oven (JSGI-150T, JSR, Yongin City, Republic of Korea) set to 37 °C for 24 h. A digital scale (AS 220/C/2, Radwag, Radom, Poland) with 0.0001 g sensitivity was used to weigh the samples after the second desiccator was maintained at 24 °C for 1 h. Except for removing and changing samples, the desiccator remained closed throughout the process. Once all samples were weighed, the silica gel in the first desiccator was replaced with freshly dried gel. Each sample’s mass was kept within 0.2 mg between successive weighings by repeating the cycle until a constant mass, referred to as the conditioned mass M1, was reached. Subsequently, the volume of each sample was calculated by averaging one center measurement, five thickness measurements, and three diameter measurements taken at evenly spaced points around the sample’s circumference.

B. Wetting samples

For seven days, the samples were placed in a water bath and then transferred to an oven set at 37 °C. Afterward, the samples were removed from the solution, dried with a clean towel, and waved around for 15 s. After 60 s of being taken out of the solution, the samples were weighed to determine their wet mass, M2. 

C. Reconditioned samples

The same process described earlier in the first phase was used to recondition the samples to a consistent mass. The weight of the samples is recorded as reconditioned mass, M3.

Calculation of water sorption and solubility:

The water sorption, Wsp, value can be calculated by applying the following equation:

Wsp = M2 − M3/V(1)

The water solubility, Wsl, value can be calculated by applying the following equation:

Wsl = M1 − M3/V(2)

V is the sample’s volume in cubic millimeters, M1 is the sample’s conditioned mass in micrograms, M2 is the sample’s wet mass in micrograms, and M3 is the sample’s reconditioned mass in micrograms.

#### 2.3.2. Surface Roughness

The three-dimensional (3D) profilometer (Contour-GT-X, 3D Optical Microscope, Bruker Nano Surfaces Division, San Jose, CA, USA) was used to measure the surface roughness (SR) of each specimen before and after polishing in micrometers (μm). For each sample, a 3D profiling device was employed to scan its surface in three dimensions, and 3D software for operations and analysis, Vision64, developed by Bruker’s Nano Surfaces Division (San Jose, CA, USA), was used to calculate the surface roughness of the specimens.

### 2.4. Analytical or Inferential Statistics

Results were analyzed statistically using the Statistical Package for the Social Sciences (SPSS 25.0, IBM/SPSS Inc., Chicago, IL, USA). Various types of statistical analyses were performed. One-way ANOVA was used to assess significant differences among more than two normally distributed groups, using continuous data. The assumptions of normality in each group and the homogeneity of variances were verified using the Shapiro–Wilk test and Levine’s test, respectively. Moreover, the Tukey-HSD test was used as a post hoc method to adjust for multiple comparisons after a significant ANOVA test to indicate which pairs of groups differ significantly. Additionally, a paired samples t-test was used to assess the statistical significance of the difference between two dependent study groups with parametric data.

Regarding the level of significance (*p*-value) in all applied tests, the *p*-values associated with test statistics indicated the significance level at which the null hypothesis (the hypothesis of no difference) was rejected. It was set at 0.05, so *p*-values ≥ 0.05 are considered statistically non-significant, *p*-values < 0.05 are significant, and *p*-values < 0.01 are highly significant.

## 3. Results

### 3.1. Water Sorption Test

**Figure 3:**
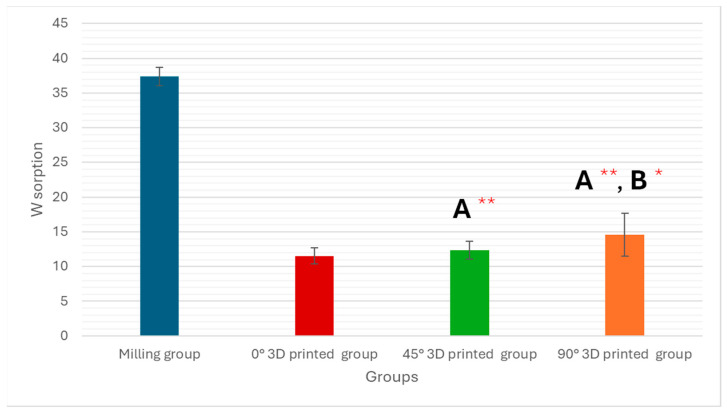
Graphic drawn of water sorption mean values ± standard deviations for all tested groups. * Statistically significant if *p* ≤ 0.05, ** highly statistically significant result if *p* ≤ 0.00, A: comparison in relation to milling technique and B: comparison in relation to 0° 3D-printed technique.

One-way ANOVA showed a significant difference among milled and three different angles (0°, 45°, and 90°) of 3D-printed groups (*p* < 0.001) in the water sorption test, as shown in Table 1 and Figure 3.

### 3.2. Water Solubility Test

**Figure 4:**
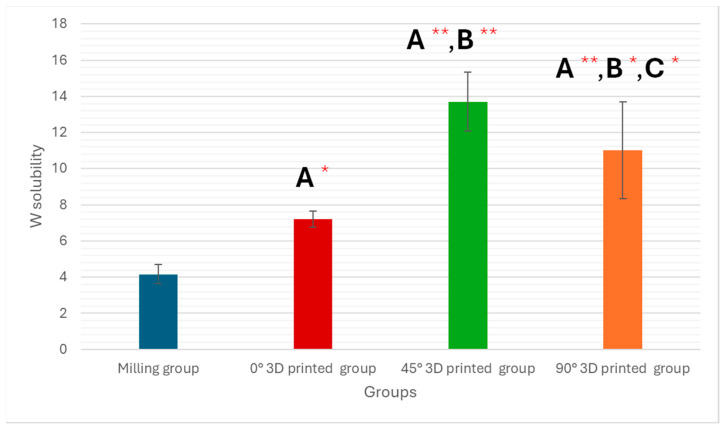
Graphic drawn of water solubility mean values ± standard deviations for all tested groups. * Statistically significant if *p* ≤ 0.05, ** highly statistically significant result if *p* ≤ 0.00, A: comparison in relation to milling technique, B: comparison in relation to 0° 3D-printed technique, and C: comparison in relation to 45° 3D-printed technique.

A one-way ANOVA showed a significant difference among milled and three specific angles (0°, 45°, and 90°) of the 3D-printed groups (*p* < 0.001) in the water solubility test, as shown in Table 2 and Figure 4.

### 3.3. Surface Roughness Test

**Figure 5:**
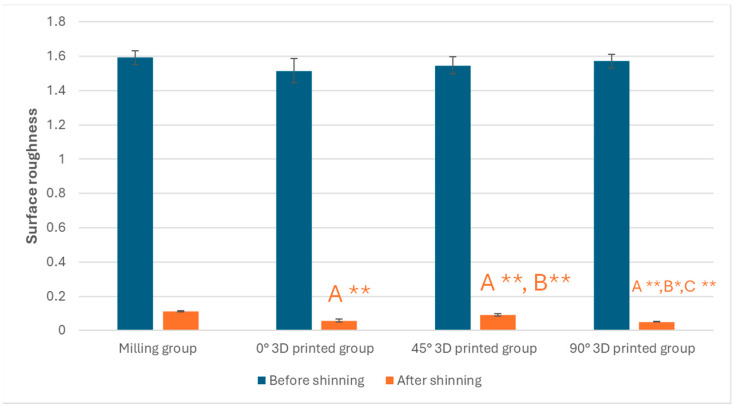
Graphic drawn of surface roughness mean values ± standard deviations for all tested groups. * Statistically significant if *p* ≤ 0.05, ** highly statistically significant result if *p* ≤ 0.00, A: comparison in relation to milling technique, B: comparison in relation to 0° 3D-printed technique, and C: comparison in relation to 45° 3D-printed technique.

The descriptive data for the various groups examined are presented in Table 3. Additionally, a paired *t*-test revealed a significant difference between the polished and unpolished versions of the milled and 3D-printed objects at three different angles (0°, 45°, and 90°) (*p* < 0.001). Furthermore, significant differences between milled and 3D-printed groups at these angles (0°, 45°, and 90°) were observed after polishing (*p* < 0.001). Before polishing, a significant difference was also found between milled and 3D-printed groups at a single angle (0°) (*p* = 0.04) (Figure 5).

## 4. Discussion

This research examined the physical characteristics of hybrid resin composite restoration materials produced using CAD/CAM software with subtractive and additive manufacturing methods. The physical parameters analyzed included surface roughness, water solubility, and water sorption. It can now declare that the study’s hypothesis was rejected. Based on the current study’s findings, comparing CAD/CAM-fabricated hybrid resin composite restorations made by subtractive and additive processes showed significant differences.

Water sorption testing was conducted on hybrid resin composite materials created through additive and subtractive manufacturing processes in this study. Significant differences were observed in water sorption and water solubility tests between the milling group and the three different orientations (0°, 45°, and 90°) of the 3D-printed groups, indicating that the additive manufacturing method yielded unique results. 

According to the ISO 4049:2009 criteria, which state that polymer-based restorative materials should have a maximum sorption capacity of 40 μg/mm^3^ [22], this study assessed the water sorption of the examined materials. The water sorption standards set by the ISO were met by the CAD/CAM and 3D-printed materials tested. Restorative materials are known to degrade when they absorb excessive amounts of water, leading to the deterioration of the resin matrix and elution of monomers and other degradation products [23].

It has been argued that water diffusion decreases as filler content increases because the polymer matrix content decreases [24,25,26]. The results of this study agree that 3D-printed resin composites with the lowest filler percentage showed significantly higher water sorption than milled resin composites (*p* < 0.001). However, this study also found that milled resin composites had high average water sorption values. This suggests that the composition, including the filler and resin amounts, may have a greater influence on water sorption than the manufacturing method [24]. Therefore, it is reasonable to think that water sorption is proportional to the percentage of the polymer matrix [25].

Water solubility is influenced by many factors. According to ISO 4049, the water solubility value of hybrid resin composites should not exceed 7.5 μg/mm^3^ [22]. Hybrid resin composites may fail due to water solubility, which can affect the dimensional stability of the prosthesis [27,28,29]. Conversely, if the composites are very soluble, they will contain more unreacted monomers, which can harm oral tissues. Minimizing solubility and water sorption is crucial for a successful material [30]. Several studies [15,19] have examined the solubility and sorption of 3D-printed temporary resins. Photopolymer and 3D-printed polymethylmethacrylate (PMMA) provisional resins performed better than CAD/CAM-milled PMMA and traditional bis-acryl resins in terms of solubility and water sorption, while conventional PMMA resins showed lower water sorption. Researcher Perea-Lowery et al. [31] found that the polymerization method contributed to the high solubility and water sorption of 3D-printed resins. Due to the multi-layer printing process, 3D-printed materials are prone to dimensional changes caused by water infiltrating between the layers and moving the polymer chains. Additionally, the lower degree of polymerization in 3D-printed materials [31], which results in free monomers, further increases their water sorption [28].

The degree of conversion, the structure of the monomers, and fillers are all factors affecting the water solubility of hybrid resin composites [32]. Water solubility in these composites is indicated by the amount of water-soluble components, unreacted monomers, plasticizers, and initiators that leach out when the specimens are submerged in water [33,34]. Materials with higher solubility will absorb more water, as observed by Lassila and Vallittu [34], who noted a correlation between water sorption and solubility in resin composites. In this study, the increased water solubility in the 3D-printed groups may be due to differences in chemical composition between 3D-printed and milled resin composites [35]. The 3D-printed resin composite (CROWNTEC resin) used here contains a Bis-EMA matrix monomer [36]. The hydrophilic characteristics of these monomers are lower than those of the TEGDMA found in milled ceramics (SHOFU DISK HC) [36]. The reduced water solubility of the milled resin composite may be attributed to its hydrophobic monomer content, according to this study [36].

The physical characteristics of printed specimens improve when the orientation is set to 0°, as this requires fewer layers, and the force is perpendicular to the printing layers [37]. Since different printing orientations yield different results, choosing the right orientation is crucial for achieving the desired qualities in the final product. The properties of 3D-printed resin are also influenced by the layer thickness; a thinner layer leads to a higher degree of conversion [31]. To enhance the qualities of 3D-printed resin, the amount of uncured monomer decreases as the degree of polymerization increases [38]. Temperature and post-curing time are additional factors that can boost the degree of polymerization, with a higher temperature and longer post-curing times improving the physico-mechanical properties of 3D-printed resins [38]. Therefore, adjusting printing parameters can optimize the qualities of 3D-printed resins for creating the best prosthesis [39]. Multiple studies emphasize that the printing direction significantly affects the material’s physical properties, making 0 degrees the preferred orientation over other angles.

Regarding surface roughness, 3D-printed resin composites showed the lowest values compared to milled resin composites, both before and after polishing. These findings can be explained by the fact that the evaluated composite-based materials have different microstructural compositions. It is expected that 3D-printed resins will exhibit structural stability and fluid consistency during printing and storage. Permanent restorations made from 3D-printed composite resin materials, similar to flowable dental composites, should, to maintain their liquid consistency, contain fewer inorganic fillers than CAD/CAM composite blocks. The mechanical properties of the composite material are also influenced by a low filler content [40]. According to Kumari et al. [41], surface roughness can be reduced as the filler fraction increases. Surface roughness values indicate that 3D-printed composite resins have less roughness than CAD/CAM resin blocks examined in the current study, even though the 3D-printed composites tested had a lower filler content compared to CAD/CAM-milled composites.

Surface roughness values improved in both additive and subtractive groups after polishing [42]. It should be noted that well-executed polishing procedures can reduce roughness parameter values; however, the final surface roughness can still be influenced by material composition factors such as the size, ratio, type of inorganic filler, and structure of the organic matrix [23,43].

Furthermore, the clinical implications of these findings warrant consideration. While subtractive manufacturing demonstrated superior physical properties in this in vitro setting, the advantages of additive manufacturing, such as reduced material waste, potential for chairside fabrication, and geometric design flexibility, cannot be overlooked [35]. The ability to customize restorations with complex internal features or precisely match shade gradients may offer clinical advantages not readily achievable with traditional milling [35]. However, realizing the full potential of 3D-printed restorations necessitates the careful optimization of printing parameters, post-processing techniques, and material formulations to ensure acceptable mechanical properties, long-term durability, and biocompatibility.

The findings of this study underscore the importance of considering both the manufacturing method and the specific material composition when selecting restorative materials. While the milled hybrid resin composite demonstrated superior physical properties in the short-term, the long-term performance of these materials under simulated oral conditions remains to be fully elucidated. Factors such as cyclic loading, thermal cycling, and exposure to various oral fluids can significantly influence the degradation and failure of dental restorations. Therefore, future studies should incorporate long-term testing protocols to assess the durability and clinical relevance of both CAD/CAM-milled and 3D-printed hybrid resin composites, taking into account the potential for water sorption, solubility, and surface roughness to affect their structural integrity and biocompatibility over time. Additionally, future research should focus on developing novel resin composites specifically tailored for additive manufacturing, as well as exploring advanced post-curing methods to enhance the mechanical properties and reduce the water sorption and solubility of 3D-printed dental restorations [42].

One drawback of this study is that it only used one type of hybrid ceramic material. However, the study could have been improved by including different hybrid resin composite materials and comparing them through 3D printing and subtractive methods. Therefore, more comprehensive results might be achieved in future research that explores other combinations.

## 5. Conclusions

This study quantitatively demonstrated that the manufacturing technique and printing orientation significantly affect the physical properties of hybrid resin composite materials. Milled specimens exhibited a superior performance compared to all 3D-printed groups, with statistically lower water sorption (37.37 ± 1.35 µg/mm^3^) and water solubility (4.16 ± 0.52 µg/mm^3^) values. Among the 3D-printed groups, the 0° orientation showed the most favorable outcomes, with the lowest water sorption (11.52 ± 1.12 µg/mm^3^) and solubility (7.21 ± 0.45 µg/mm^3^) among printed samples. Surface roughness measurements confirmed that polishing significantly improved surface quality in all groups, reducing roughness in milled samples from 1.592 ± 0.039 µm to 0.112 ± 0.002 µm, and in 0° 3D-printed samples from 1.515 ± 0.071 µm to 0.058 ± 0.010 µm. These results underscore the importance of post-processing and highlight the clinical potential of 3D-printed hybrid composites when optimized printing parameters and finishing protocols are applied. In conclusion, subtractive manufacturing yielded better overall performance, but 3D printing at a 0° orientation, followed by proper polishing, produced comparable surface characteristics and may offer a viable alternative with cost and material-saving advantages.

## Figures and Tables

**Table 1:** Analysis of water sorption within the study groups.

Variables	Study Groups				Significance Test
	Milling (n = 5)	0° 3D-printed (n = 5)	45° 3D-printed (n = 5)	90° 3D-printed (n = 5)	
**Water Sorption**	37.37 ± 1.35	11.52 ± 1.12	12.32 ± 1.27	14.56 ± 3.11	F = 212.688 *p* < 0.001 **
**P_1_**		<0.001 **	<0.001 **	<0.001 **	
**P_2_**			0.512	0.022 *	
**P_3_**				0.080	

Data are expressed as mean ± SD, F for ANOVA test, * statistically significant if *p* ≤ 0.05, ** highly statistically significant result if *p* ≤ 0.00, P_1_: comparison in relation to milling technique, P_2_: comparison in relation to 0° 3D-printed technique, and P_3_: comparison in relation to 45° 3D-printed technique.

**Table 2:** Analysis of water solubility within the study groups.

Variables	Study Groups				Significance Test
	Milling (n = 5)	0° 3D-printed (n = 5)	45° 3D-printed (n = 5)	90° 3D-printed (n = 5)	
**Water Solubility**	4.16 ± 0.52	7.21 ± 0.45	13.70 ± 1.63	11.01 ± 2.69	F = 33.856 *p* < 0.001 **
**P_1_**		0.009 *	<0.001 **	<0.001 **	
**P_2_**			<0.001 **	0.002 *	
**P_3_**				0.018 *	

Data are expressed as mean ± SD, F for ANOVA test, * statistically significant if *p* ≤ 0.05, ** highly statistically significant result if *p* ≤ 0.00, P_1_: comparison in relation to milling technique, P_2_: comparison in relation to 0° 3D-printed technique, and P_3_: comparison in relation to 45° 3D-printed technique.

**Table 3:** Analysis of surface roughness within the study groups.

Variables	Study Groups				Significance Test
	Milling (n = 10)	0° 3D-printed (n = 10)	45° 3D-printed (n = 10)	90° 3D-printed (n = 10)	
**Before Polishing (Shinning)**					
**Surface Roughness**	1.592 ± 0.039	1.515 ± 0.071	1.546 ± 0.050	1.571 ± 0.041	F = 3.652 *p* = 0.023 *
**P_1_**		0.004 *	0.072	0.393	
**P_2_**			0.210	0.030 *	
**P_3_**				0.326	
**After Polishing (Shinning)**					
**Surface Roughness**	0.112 ± 0.002	0.058 ± 0.010	0.090 ± 0.007	0.050 ± 0.002	F = 195.196 *p* < 0.001 **
**P_1_**		<0.001 **	<0.001 **	<0.001 **	
**P_2_**			<0.001 **	0.013 *	
**P_3_**				<0.001 **	
**Comparison (before and after polishing)** **(Shinning)**	t = 110.483 *p* < 0.001 **	t = 61.583 *p* < 0.001 **	t = 89.606 *p* < 0.001 **	t = 108.441 *p* < 0.001 **	

Data are expressed as mean ± SD, F for ANOVA test, * statistically significant if *p* ≤ 0.05, ** highly statistically significant result if *p* ≤ 0.00, P_1_: comparison in relation to milling technique, P_2_: comparison in relation to 0° 3D-printed technique, and P_3_: comparison in relation to 45° 3D-printed technique.

## Data Availability

The original contributions presented in this study are included in the article. Further inquiries can be directed to the corresponding author.

## References

[1] Dawood A., Marti B.M., Sauret-Jackson V., Darwood A. (2015). 3D printing in dentistry. Br. Dent. J..

[2] Abduo J., Lyons K., Bennamoun M. (2014). Trends in computer-aided manufacturing in prosthodontics: A review of the available streams. Int. J. Dent..

[3] Moon W., Kim S., Lim B.-S., Park Y.-S., Kim R.J.-Y., Chung S.H. (2021). Dimensional accuracy evaluation of temporary dental restorations with different 3D printing systems. Materials.

[4] Kantaros A., Ganetsos T., Piromalis D. (2023). 3D and 4D printing as integrated manufacturing methods of industry 4.0. Am. J. Eng. Appl. Sci..

[5] Grzebieluch W., Kowalewski P., Grygier D., Rutkowska-Gorczyca M., Kozakiewicz M., Jurczyszyn K. (2021). Printable and machinable dental restorative composites for CAD/CAM application—Comparison of mechanical properties, fractographic, texture and fractal dimension analysis. Materials.

[6] Daher R., Ardu S., di Bella E., Krejci I., Duc O. (2024). Efficiency of 3D-printed composite resin restorations compared with subtractive materials: Evaluation of fatigue behavior, cost, and time of production. J. Prosthet. Dent..

[7] Prause E., Malgaj T., Kocjan A., Beuer F., Hey J., Jevnikar P., Schmidt F. (2024). Mechanical properties of 3D-printed and milled composite resins for definitive restorations: An in vitro comparison of initial strength and fatigue behavior. J. Esthet. Restor. Dent..

[8] Elkaffas A.A., Eltoukhy R.I., Elnegoly S.A., Mahmoud S.H. (2022). 36-Month Randomized Clinical Trial Evaluation of Preheated and Room Temperature Resin Composite. Oper. Dent..

[9] Coldea A., Swain M.V., Thiel N. (2013). Mechanical properties of polymer-infiltrated-ceramic-network materials. Dent. Mater..

[10] Coldea A., Swain M.V., Thiel N. (2013). In-vitro strength degradation of dental ceramics and novel PICN material by sharp indentation. J. Mech. Behav. Biomed. Mater..

[11] Wendler M., Belli R., Valladares D., Petschelt A., Lohbauer U. (2018). Chairside CAD/CAM materials. Part 3: Cyclic fatigue parameters and lifetime predictions. Dent. Mater..

[12] Bondarenko A.V., Islamov S.R., Ignatyev K.V., Mardashov D.V. (2020). Laboratory investigation of polymer compositions for ell killing in fractured reservoirs. Perm J. Pet. Min. Eng..

[13] Belousov A., Lushpeev V., Sokolov A., Sultanbekov R., Tyan Y., Ovchinnikov E., Shvets A., Bushuev V., Islamov S. (2025). Experimental Research of the Possibility of Applying the Hartmann–Sprenger Effect to Regulate the Pressure of Natural Gas in Non-Stationary Conditions. Processes.

[14] Eldafrawy M., Nguyen J.-F., Mainjot A., Sadoun M. (2018). A functionally graded PICN material for biomimetic CAD-CAM blocks. J. Dent. Res..

[15] Shin J.-W., Kim J.-E., Choi Y.-J., Shin S.-H., Nam N.-E., Shim J.-S., Lee K.-W. (2020). Evaluation of the color stability of 3D-printed crown and bridge materials against various sources of discoloration: An in vitro study. Materials.

[16] Yao Q., Morton D., Eckert G.J., Lin W.-S. (2021). The effect of surface treatments on the color stability of CAD-CAM interim fixed dental prostheses. J. Prosthet. Dent..

[17] Atria P.J., Lagos I., Sampaio C.S. (2020). In vitro evaluation of surface roughness, color stability, and color masking of provisional restoration materials for veneers and crowns. Int. J. Comput. Dent..

[18] Taşın S., Ismatullaev A., Usumez A. (2022). Comparison of surface roughness and color stainability of 3-dimensionally printed interim prosthodontic material with conventionally fabricated and CAD-CAM milled materials. J. Prosthet. Dent..

[19] Song S.-Y., Shin Y.-H., Lee J.-Y., Shin S.-W. (2020). Color stability of provisional restorative materials with different fabrication methods. J. Adv. Prosthodont..

[20] Goodacre B.J., Goodacre C.J., Baba N.Z., Kattadiyil M.T. (2018). Comparison of denture tooth movement between CAD-CAM and conventional fabrication techniques. J. Prosthet. Dent..

[21] Özcan C., Lestriez P., Berry-Kromer V., Thiébaud F., Sockalingum G., Untereiner V., Angiboust J.-F., Josset Y. (2020). Misinterpretation of ISO 4049 standard recommendations: Impact on Young’s modulus and conversion degree of dental composites. J. Mech. Behav. Biomed. Mater..

[22] (2009). Dentistry: Polymer-Based Restorative Materials.

[23] Alhassan M., Maawadh A., Labban N., Alnafaiy S.M., Alotaibi H.N., BinMahfooz A.M. (2022). Effect of different surface treatments on the surface roughness and gloss of resin-modified CAD/CAM ceramics. Appli Sci..

[24] Ling L., Ma Y., Malyala R. (2021). A novel CAD/CAM resin composite block with high mechanical properties. Dent. Mater..

[25] Alamoush R.A., Salim N.A., Silikas N., Satterthwaite J.D. (2022). Long-term hydrolytic stability of CAD/CAM composite blocks. Eur. J. Oral Sci.

[26] Alshali R.Z., Salim N.A., Satterthwaite J.D., Silikas N. (2015). Long-term sorption and solubility of bulk-fill and conventional resin-composites in water and artificial saliva. J. Dent..

[27] Figuerôa R.M.S., Conterno B., Arrais C.A.G., Sugio C.Y.C., Urban V.M., Neppelenbroek K.H. (2018). Porosity, water sorption and solubility of denture base acrylic resins polymerized conventionally or in microwave. J. Appl. Oral Sci.

[28] Berli C., Thieringer F.M., Sharma N., Müller J.A., Dedem P., Fischer J., Rohr N. (2020). Comparing the mechanical properties of pressed, milled, and 3D-printed resins for occlusal devices. J. Prosthet. Dent..

[29] Gad M.M., Alshehri S.Z., Alhamid S.A., Albarrak A., Khan S.Q., Alshahrani F.A., Alqarawi F.K. (2022). Water sorption, solubility, and translucency of 3D-printed denture base resins. Dent. J..

[30] Machado C., Rizzatti-Barbosa C.M., Gabriotti M.N., Joia F.A., Ribeiro M.C., Sousa R.L. (2004). Influence of mechanical and chemical polishing in the solubility of acrylic resins polymerized by microwave irradiation and conventional water bath. Dent. Mater..

[31] Perea-Lowery L., Gibreel M., Vallittu P.K., Lassila L.V. (2021). 3D-printed vs. heat-polymerizing and autopolymerizing denture base acrylic resins. Materials.

[32] Misilli T., Gönülol N. (2017). Water sorption and solubility of bulk-fill composites polymerized with a third generation LED LCU. Braz. Oral Res..

[33] Cucci A.L.M., Vergani C.E., Giampaolo E.T., Afonso M.C.d.S.F. (1998). Water sorption, solubility, and bond strength of two autopolymerizing acrylic resins and one heat-polymerizing acrylic resin. J. Prosthet. Dent..

[34] Lassila L., Vallittu P. (2001). Denture base polymer Alldent Sinomer^®^: Mechanical properties, water sorption and release of residual compounds. J. Oral Rehabil..

[35] Alanazi K.K., Alzaid A.A., Elkaffas A.A., Bukhari S.A., Althubaitiy R.O., Alfaifi K.A., Alfahdi I.M., Alqahtani H.A. (2024). Mechanical Assessment of CAD/CAM Fabricated Hybrid Ceramics: An In Vitro Study. Appl. Sci..

[36] Gajewski V.E., Pfeifer C.S., Fróes-Salgado N.R., Boaro L.C., Braga R.R. (2012). Monomers used in resin composites: Degree of conversion, mechanical properties and water sorption/solubility. Braz. Dent. J..

[37] Shim J.S., Kim J.-E., Jeong S.H., Choi Y.J., Ryu J.J. (2020). Printing accuracy, mechanical properties, surface characteristics, and microbial adhesion of 3D-printed resins with various printing orientations. J. Prosthet. Den..

[38] Aati S., Akram Z., Shrestha B., Patel J., Shih B., Shearston K., Ngo H., Fawzy A. (2022). Effect of post-curing light exposure time on the physico–mechanical properties and cytotoxicity of 3D-printed denture base material. Dent. Mater..

[39] Bayarsaikhan E., Lim J.-H., Shin S.-H., Park K.-H., Park Y.-B., Lee J.-H., Kim J.-E. (2021). Effects of postcuring temperature on the mechanical properties and biocompatibility of three-dimensional printed dental resin material. Polymers.

[40] Ang S.F., Scholz T., Klocke A., Schneider G.A. (2009). Determination of the elastic/plastic transition of human enamel by nanoindentation. Dent. Mater..

[41] Kumari C.M., Bhat K.M., Bansal R. (2016). Evaluation of surface roughness of different restorative composites after polishing using atomic force microscopy. J. Conserv. Dent..

[42] Robaian A., Alshehri A.M., Alqhtani N.R., Almudahi A., Alanazi K.K., Abuelqomsan M.A., Raffat E.M., Elkaffas A., Hashem Q., Soliman T.A. (2025). Effect of Surface Treatments and Thermal Aging on Bond Strength Between Veneering Resin and CAD/CAM Provisional Materials. Polymers.

[43] Elkaffas A.A., Alshehri A.M., Alqahtani A.R., Albaijan R.S., Soliman T.A. (2024). Impact of Various Cavity-Preparation Designs on Fracture Resistance and Failure Mode of CAD/CAM Fabricated Ceramic Inlays and Onlays. Appl. Sci..

